# Freeze the clock: earlier catheter ablation for atrial fibrillation delivers better outcomes

**DOI:** 10.1093/europace/euaf010

**Published:** 2025-01-21

**Authors:** Marco Schiavone, Luigi Di Biase

**Affiliations:** Department of Clinical Electrophysiology and Cardiac Pacing, Centro Cardiologico Monzino, IRCCS, Milan, Italy; Cardiac Arrhythmia Center, Division of Cardiology, Montefiore-Einstein Center, Albert Einstein College of Medicine, 111 East 210 Street, Bronx, NY 10467, USA

**This editorial refers to ‘Impact of atrial fibrillation diagnosis-to-ablation time on 24-month efficacy and safety outcomes in the Cryo Global Registry’ by D. Lawin *et al.*, https://doi.org/10.1093/europace/euaf008.**

The prevalence of atrial fibrillation (AF) is projected to rise substantially in the coming years, imposing a considerable burden on healthcare systems.^1^ The rhythm control strategy remains a critical aspect of clinical practice.^2^ Early intervention in AF appears beneficial, as prolonged AF leads to progressive atrial fibrosis and remodelling, which in turn reduces the efficacy of antiarrhythmic drug (AAD) therapies.^3^ Recent evidence indicates that catheter ablation (CA) achieves higher rates of freedom from AF recurrences compared with AADs in patients with paroxysmal AF (PAF).^4^ The EAST-AFNET-4 trial highlighted this shift, demonstrating that early rhythm control significantly reduces composite cardiovascular outcomes [3.9 vs. 5.0 events per 100 person-years; hazard ratio (HR), 0.79; 96% confidence interval (CI), 0.66–0.94, *P* = 0.005].^5^ Notably, only a small number (8%) of patients in this trial initially underwent CA, with the proportion increasing to 19.4% by the trial’s end. Additionally, first-line CA in PAF patients lowers the progression risk to persistent AF (PsAF).^6^ Despite these findings, CA is often delayed due to concerns about procedure-related adverse events. Recent observational studies show that postponing CA may increase morbidity, with up to 19.2% of patients on waitlists experiencing significant adverse outcomes.^7^

Cryoballoon ablation (CBA) has consistently shown good outcomes after pulmonary vein isolation (PVI) for PAF, even regardless of the presence of PV variants.^8^ In the EARLY-AF trial,^9^ early CBA achieved significantly lower rates of arrhythmia recurrence compared with AADs (42.9% vs. 67.8%; HR, 0.48; 95% CI, 0.35–0.66; *P* < 0.001) over a 1-year follow-up. The STOP-AF^10^ trial corroborated these findings, showing that early CBA was superior to AADs in achieving treatment success at 12 months (74.6% vs. 45.0%; *P* < 0.001). Before the adoption of CBA, radiofrequency ablation (RFA) had already demonstrated the potential benefits of early ablation in AF management. The RAAFT and RAAFT-2 trials established the feasibility of early RFA as a first-line treatment, showing significant reductions in AF recurrences and improved clinical outcomes in managing PAF.^11^

In this issue of *Europace*, Lawin *et al.*^12^ investigated the efficacy and safety of CBA performed within 12 months of AF diagnosis (early ablation) vs. after 12 months (late ablation) in a sub-study from the Cryo-AF Global Registry. This real-world evaluation provided a robust contribution to the growing body of evidence supporting early CBA, and all investigators should be commended for their work. The authors concluded that early CBA resulted in higher freedom from arrhythmic recurrence and lower periprocedural complications. Notably, this study's strength lies in its multicenter patients’ enrolment, including 3447 subjects across 37 countries. By leveraging this large and diverse cohort of both PAF and PsAF patients (28.5% vs. 25.0% PsAF patients in early-ablation vs. late-ablation groups), the authors demonstrated that early ablation was associated with a 33% lower risk of arrhythmic recurrence compared with delayed intervention. Their comprehensive analysis extends beyond arrhythmia outcomes, addressing quality-of-life improvements. The EQ-5D index score for overall quality of life significantly improved from baseline to 12 and 24 months in both cohorts, without differences among groups. Early CBA was linked to smaller left atrial diameters and better baseline cardiovascular health, suggesting that timely intervention prevents the progression of atrial cardiomyopathy and preserves ablation outcomes before atrial remodelling occurs. These findings align with prior research that underscores the importance of addressing AF as early as possible. Indeed, both the STOP-AF^9^ and EARLY-AF^10^ trials highlighted that early intervention could curb the progression to PsAF, a critical outcome supported by Lawin *et al.*^12^ as well. The STOP-AF trial^9^ demonstrated that ablation significantly reduced progression to PsAF (1.9% vs. 7.4% over 3 years). This finding is particularly relevant with CBA, as PsAF is associated with worse ablation outcomes than PAF after PVI-only procedures.

In addition real-world data further support early ablation. The Danish Nationwide Register study by Tønnesen *et al.*^13^ demonstrated a significant reduction in AF recurrence with ablation within 1 year of diagnosis compared with delayed intervention (5-year cumulative recurrence: 42.9% for early ablation vs. 58.4% for ablation > 2.9 years after diagnosis; HR for late ablation, 1.40; 95% CI, 1.28–1.53). Additionally, in this study, the authors reported that early ablation reduced major adverse cardiovascular events, including heart failure and stroke, compared with late ablation. Unfortunately, no comparisons could be made with the study by Lawin *et al.*^12^ as it did not evaluate cardiovascular mortality or other hard cardiovascular endpoints. Data from Erhard *et al.*^14^ provide additional granularity, extending the benefits of early ablation even in PsAF, specifically in younger patients ≤ 55 years. In their study, ablation within 12 months of diagnosis halved the recurrence rate compared with delayed ablation (25.5% vs. 52.0%; *P* = 0.003), emphasizing the importance of early intervention not only to delay progression from PAF to PsAF but also to achieve better outcomes even when AF has already advanced to a persistent stage.

Despite the compelling evidence favouring early CA, some studies have questioned its long-term superiority. For instance, Kalman *et al.*^15^ found no significant difference in arrhythmia-free survival at 12 months between early (within 1 month) and delayed ablation (optimized medical therapy followed by CA at 12 months post-recruitment) strategies (56.3% vs. 58.6%; HR, 1.12; 95% CI, 0.59–2.13; *P* = 0.7). This discordance is likely attributed to differences in patient populations, AF burden, and procedural variability. However, there are several aspects of this study that warrant further consideration, particularly as some findings appear to contrast with existing literature. First, the relatively small sample size (100 patients) limited its statistical power, making it less sensitive than the Cryo-AF Global Registry subanalysis^12^ to detect subtle differences in outcomes. Second, the small number of randomized patients prevented the investigators from balancing baseline patient characteristics, resulting in a numerically higher proportion of patients with PAF in the delayed ablation group. This imbalance may have biased the results in favour of the delayed strategy. Additionally, differences in follow-up durations, not fully accounted for by the study design, may have introduced bias in the assessment of long-term outcomes.

Concerns about procedural risks have historically deterred early referrals for ablation. However, recent data counter these fears, at least partially. Lawin *et al.*^12^ reported lower rates of serious adverse events in the early ablation cohort compared with late ablation (2.4% vs. 3.5%; *P* = 0.045). Although the overall complication rate remains non-negligible, 14.2% of complications were identified as phrenic nerve palsy, a technique-specific issue likely to be mitigated with the adoption of non-thermal ablation methods (such as pulsed field ablation), while 37.3% involved groin complications, such as hematomas, not always requiring specific treatment and mitigated by the progressive adoption of echo-guided vein punctures. The overall rate of cardiac tamponade, perforation, and pericardial effusion was 0.4%; however, intracardiac echocardiography was utilized in only a small proportion of patients (27.5%). If the rate of periprocedural complications can be attenuated through technological advancements and increased operator experience, complications related to AADs should not be underestimated and are less likely to diminish in the future. While a direct comparison between these two strategies is beyond the scope of the Cryo-AF Global Registry, the STOP-AF^9^ and EARLY-AF^10^ trials reported comparable safety profiles between CBA and AADs, with serious adverse events occurring in 3.2% of patients in the ablation group (consistent with the findings of Lawin *et al.*^12^) and 4.0% in the AAD group in EARLY-AF.^10^ The lower rate of serious adverse events in the early ablation cohort in the study by Lawin *et al.*^12^ is particularly reassuring, countering concerns that early intervention might pose heightened procedural risks or carry a higher risk than AADs. The better safety profile of early CA is likely attributable to early ablation patients being generally healthier, with fewer comorbidities and smaller left atrial diameters-factors that independently contribute to improved outcomes in both efficacy and safety. Adverse events associated with the continuous use of AADs tend to increase over time, posing greater risks for younger patients.

While this study^12^ presents compelling data, certain limitations merit attention. The non-randomized design introduces potential biases, particularly regarding patient selection and baseline differences. Future randomized trials with stratified patient populations are essential to definitively address these confounding factors. Moreover, questions about the long-term prognostic benefits of early ablation on mortality and major adverse cardiovascular events remain. Although the EAST-AFNET-4 trial^5^ demonstrated improvements in cardiovascular endpoints with early rhythm control, only a minority of its participants underwent ablation, and the evaluation of these endpoints was beyond the scope of the study under discussion.

This report by Lawin *et al.*^12^ strengthens the evidence supporting early CA as a cornerstone in AF management, particularly within the first year of diagnosis. While detractors of early ablation may argue that its better outcomes are due to patients being healthier or younger, it is important to note that patients often become older and sicker while waiting for CA on prolonged waitlists or delaying ablation. Clinicians should be encouraged to refer patients for ablation early, rather than deferring until symptoms or arrhythmia burden escalate. The consistent benefits of early ablation—reduced arrhythmia recurrence, lower progression to PsAF and atrial remodelling, and fewer adverse events—support a shift in practice patterns towards prioritizing timely referrals.

## Data Availability

Data sharing is not applicable to this article as no new data were created or analysed in this study.
